# Understanding the Dependence of Nanoparticles Magnetothermal Properties on Their Size for Hyperthermia Applications: A Case Study for La-Sr Manganites

**DOI:** 10.3390/nano11071826

**Published:** 2021-07-14

**Authors:** Mylla C. Ferreira, Bruno Pimentel, Vivian Andrade, Vladimir Zverev, Radel R. Gimaev, Andrei S. Pomorov, Alexander Pyatakov, Yulia Alekhina, Aleksei Komlev, Liudmila Makarova, Nikolai Perov, Mario S. Reis

**Affiliations:** 1 Institute of Physics, Fluminense Federal University, Niterói 24210-346, RJ, Brazil; myllacf@id.uff.br (M.C.F.); brunompim@gmail.com (B.P.); 2 IFIMUP—Institute of Physics for Advanced Materials, Nanotechnology and Photonics of University of Porto, 4169-007 Porto, Portugal; viviancamposandrade@gmail.com; 3 ”Gleb Wataghin” Physics Institute, State University of Campinas, Campinas 13083-859, SP, Brazil; 4 Faculty of Physics, Moscow State University, 119991 Moscow, Russia; vi.zverev@physics.msu.ru (V.Z.); gimaev@physics.msu.ru (R.R.G.); pomorov.as14@physics.msu.ru (A.S.P.); pyatakov@physics.msu.ru (A.P.); ya.alekhina@physics.msu.ru (Y.A.); komlev.as16@physics.msu.ru (A.K.); la.loginova@physics.msu.ru (L.M.); perov@magn.ru (N.P.)

**Keywords:** magnetic hyperthermia, superparamagnetism, magnetic nanoparticles, heating efficiency, mean nanoparticle size

## Abstract

Magnetic oxides are promising materials for alternative health diagnoses and treatments. The aim of this work is to understand the dependence of the heating power with the nanoparticle (NP) mean size, for the manganite composition La_0.75_Sr_0.25_MnO_3_ (LSMO)—the one with maximum critical temperature for the whole La/Sr ratio of the series. We have prepared four different samples, each one annealed at different temperatures, in order to produce different mean NP sizes, ranging from 26 nm up to 106 nm. Magnetization measurements revealed a FC-ZFC irreversibility and from the coercive field as function of temperature we determined the blocking temperature. A phase diagram was delivered as a function of the NP mean size and, based on this, the heating mechanism understood. Small NPs (26 nm) is heated up within the paramagnetic range of temperature ($T>T_{c}$), and therefore provide low heating efficiency; while bigger NPs are heated up, from room temperature, within the magnetically blocked range of temperature ($T<T_{B}$), and also provide a small heating efficiency. The main finding of this article is related with the heating process for NPs within the magnetically unblocked range of temperature ($T_{c}>T>T_{B}$), for intermediate mean diameter size of 37 nm, with maximum efficiency of heat transfer.

## 1. Introduction

Local magnetic hyperthermia is one of the promising methods for cancer treatment [1,2,3,4,5]. The therapeutic efficacy of this procedure is ensured by the possibility of local heating of the tumor by an alternating magnetic field, due to the presence of magnetic nanoparticles. The use of local hyperthermia in combination with radiation and/or chemotherapy improves the treatment of cancer patients by 20–40% [6,7]. In 1957, Gilchrist [8] used microparticles of Fe_2_O_3_ (0.02–0.1 μm) for local heating of lymph nodes in dogs, starting the history of the developments of this in vivo test method. Further studies in animals have shown, as a result of heating by magnetic nanoparticles (NPs) in an alternating magnetic field, the reduction in size and disappearance of tumors [9,10].

The ability to control the magnetic properties of NPs is of special importance. Namely, the critical temperature of NPs, in practice, avoids overheating of the magnetic fluid. This nuance is strategic from medical point of view, since overheating of a few degrees can damage living tissues. Blocking temperature (for superparamagnetic particles) depends on relaxation time and determines the conditions in which thermal fluctuation energy is comparable with magnetic anisotropy energy. It is shown in reference [11] that heating of superparamagnetic particles is more efficient than heating blocked NPs. Accordingly, sample optimization for hyperthermia applications requires the blocking temperature and the Curie temperature to be close to room temperature. This is the reason we have chosen lanthanum strontium La_0.75_Sr_0.25_MnO_3_ (LSMO) manganite for the present study, since the critical temperature of the bulk material is close to 330 K [12].

In this work, our goal is to understand how the NPs size interferes on the heating production, for hyperthermia applications. Thus, samples of LSMO with different NP were produced, in order to change the heating parameters [13]. Thus, the study of the relationship between the magnetic properties of LSMO NPs with different mean sizes and their corresponding magnetic and heating properties was delivered and thoroughly analyzed.

## 2. Experimental Techniques and Methodology

*Sample synthesis:* La_0.75_Sr_0.25_MnO_3_ (LSMO) samples were synthesized by sol-gel method (Pechini) [11,14], using: 0.0088 M Citric Acid, 0.0011 M Strontium Nitrate, 0.0033 M Lanthanum Nitrate Hexahydrate, and 0.0062 M Manganese Acetate and 2 mL of Ethylene Glycol as polymerizing agent. Considering our aim is to understand the dependence of the heating power with the NPs size, we have prepared four different samples, each one annealed at different temperatures: 600 °C, 800 °C, 900 °C and 1000 °C for 4 h in order to produce different NP sizes [15]. For the thermal treatment, 15 mL of the gel were introduced into an alumina crucible, resulting in 3 g of powder sample. These samples were produced at Fluminense Federal University—UFF.

*X-ray diffraction:* The structural characterizations were performed through X-ray diffraction using a Brucker D8 Advances, Brucker Corporation, Billerica, MA, USA (Cu-Kα = 1.54184 Å) at Fluminense Federal University—UFF. Rietveld refinements were performed using the Fullprof suite Toolbar [16].

*Microscopy:* The morphology of the nanoparticles was evaluated by Transmission Electronic Microscopy (TEM) JEOL 2100F, JEOL Ltd., Tokyo, Japan (200 kV) at Brazilian Center for Research on Physics—CBPF. We have used the software ImageJ to obtain the NP distribution.

*Magnetization:* Magnetization was measured using a SQUID magnetometer from Quantum Design at Laboratory of Materials and Low Temperatures, State University of Campinas and Faculty of Physics, Lomonosov Moscow State University—MSU.

**Figure 1:**
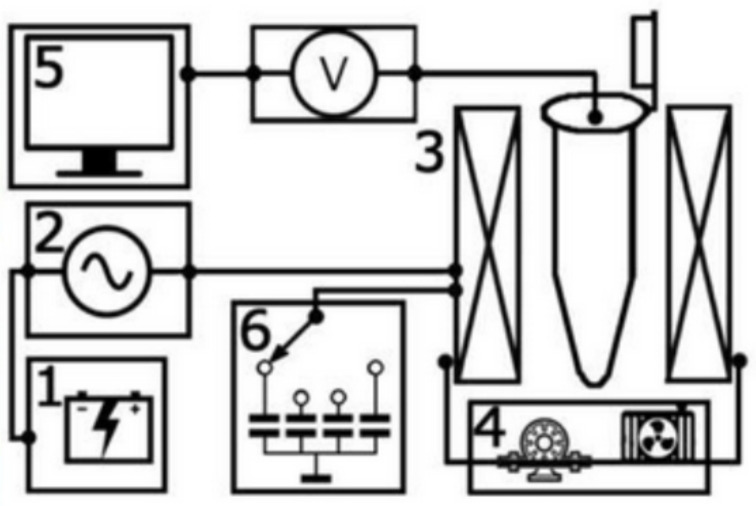
Experimental setup for heating power measurements: 1. Power supply; 2. Generator; 3. Solenoid; 4. Water cooling system; 5. System of collecting and monitoring data. 6. Re-configurable system of capacitors.

*Heating-cooling curves:* Heating power measurements were carried out at Faculty of Physics-MSU on the reconfigurable setup for high frequency magnetothermal measurements (Figure 1) made by LLC Pharmag (AMT&C Group). The AC magnetic field module was comprised of a 400 W power supply and the generator connected in series with the LC oscillating circuit, excited at resonance frequency. The LC circuit includes the reconfigurable capacitor system (to switch the resonance frequency between 150, 200, 250, and 300 kHz) and the solenoid (with the inner diameter of 7 mm, where the test-tube was placed). The temperature change of the sample is recorded using a thermocouple and a voltmeter. To avoid parasitic heating of the coil, there is a water cooling system, consisting of plastic pipes through which distilled water circulates. Heating curves represent the difference between room temperature and the temperature of the ferromagnetic fluid in the test tube (but can also be represented as the absolute value of temperature), as a function of time. The root mean square value of the AC-magnetic field can be adjusted in the intensity range from 70 Oe up to 200 Oe, by fine-tuning the frequency in the vicinity of resonance. The experimental setup is shown in Figure 1.

Since the conditions of the experiments are non-adiabatic, only the short initial fragment of the T(t) heating curve has the conditions close to adiabatic. In order to make an accurate estimate of ΔT/Δt (necessary for further evaluations), it is necessary to measure also the cooling curve to estimate the heat losses of the test tube/setup system. The adiabatic value ΔT/Δt at a certain temperature is then calculated using the *corrected slope* method, as the sum of the modules of the slope coefficients for the heating and cooling curves at this temperature [17].

In order to estimate the parasitic due to inductive heating and residual heat transfer from the coil; the heating curves of distilled water were also measured for the maximum field amplitude. This ΔT/Δt was then subtracted from the value of the corrected slope. The parasitic heating at other amplitudes was supposed to be proportional to the amplitude of the oscillating field.

## 3. Results and Discussion

### 3.1. Crystallography and Morphology

**Figure 2:**
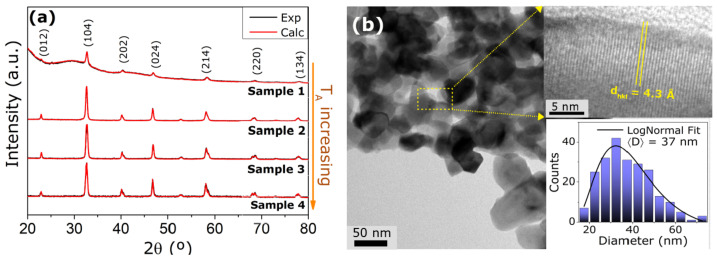
(**a**) X-ray diffraction for all samples prepared, along with the corresponding Rietveld refinement. For these fittings we have considered the space group *R-3c* related with each annealing temperature. Sample 1 presents a hump, typical of an amorphous sample. (**b**) Transmission Electronic Microscopy (TEM) images for sample 2 (37 nm) with the corresponding NP size distribution, obtained using the software ImageJ. The solid line is a LogNormal fitting curve. This morphology is representative for all samples, except for sample 1, presenting a fraction of amorphous phase. Inset of panel (**b**) also shows a magnification, revealing the fine crystalline feature of the structure. See Table 1 for a correspondence between sample label, annealing temperature and mean diameter size.

Normalized X-ray diffraction (XRD) patterns for all samples prepared and the refinement results are presented in Figure 2a. We observed that all samples crystallize in the desirable rhombohedral structure of the LSMO [18]. Sample 1 has a remarkable hump, revealing the formation of an amorphous fraction [19] (88%). As can be noted in Figure 2a, from samples 1 to 4, there is a shortening of the peaks and a shift of the Bragg positions toward higher angles, indicating a reduction of the unit cell volumes with the annealing temperature (T${}_{A}$). This behavior is due to the increase of nanoparticle size for higher heat treatment temperatures, as observed in previous mixed valence manganites nanostructures [20]. The Rietveld refinements were performed following the space group *R-3c* (# 161), with the starting lattice parameters for the bulk a=b=5.5165 Å and c= 13.3743 Å [21]. From these calculations, the lattice parameters were acquired and are summarized in Table 1. The best calculated patterns from the refinements are given by the solid red curves in Figure 2a.

From the XRD results, it is possible to estimate the mean crystallite size $D_{S}$ for each sample through the Scherrer equation:

(1) $$D_{S}=\frac{k\lambda }{\beta \cos\theta },$$

where *k* is a constant close to 1, λ is the X-ray wavelength and θ the diffraction angle of the most intense peaks. Above, β is given by: $\sqrt{U\tan^{2}\theta +V\tan\theta +W}$, where *U*, *V* and *W* are related to the full width at half maximum (FWHM) of the Pseudo-Voight function used to fit the XRD patterns [11]. The values calculated from Equation (1) for all samples analyzed are also listed in Table 1. The increment on the mean crystallite size as the annealing temperature increases is in agreement with the sharpening of XRD peaks discussed above.

In order to confirm the structural analysis, Transmission Electronic Microscopy (TEM) images were acquired for all samples (see Appendix A). Figure 2b depicts the images obtained for sample 2, revealing a rhombohedral-like morphology, with a fine level of nanostructure agglomeration, as expected from this sample route of synthesis (see Section 2 and Reference [22]). Concerning biomedical applications, nanoparticles are required to be suspended in a colloidal solution to be further coated and functionalized [23]. For this reason, further experimental studies should be performed to enable the separation of the nanoparticles through the use of chemical reactants and/or sonification, following some procedures from the literature [24,25]. Moreover, the inset of this figure presents the nanoparticle (NP) size distribution, as well the corresponding LogNormal fitting, given by [26]:

(2) $$f\left(D\right)=\frac{A}{\sigma D\sqrt{2\pi }}\exp\left\{-\frac{{\left[\ln\left(D\right)-\mu \right]}^{2}}{2\sigma^{2}}\right\}.$$

Above, *D* represents the NP size, *A* stands for the amplitude; while the parameters μ (location) and σ (scale) define the mean size of the NPs, as [26]:

(3) $$\langle D\rangle =e^{\mu +\sigma^{2}/2}.$$

Using these relations, the mean particle size was calculated for all samples prepared and are presented in Table 1. Similar to the behavior of the mean particle size obtained from XRD analysis, there is an increase of this quantity as the annealing temperature increases. Smaller nanoparticles, samples 2 and 3, present a good match with the diameter obtained from XRD analysis, suggesting that, on average, the particles are composed of a single unit of cell volume. On the other hand, for the samples annealed at higher temperatures, those two methods (Scherrer and TEM), reveal that the NPs are polycrystalline. From now on, the samples will be labeled by their NP size.

**Figure 3:**
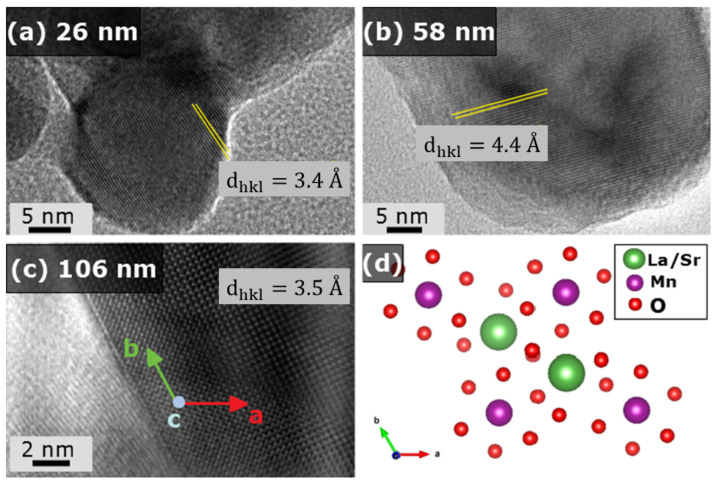
High-resolution images for the nanoparticles with (**a**) 26 nm, (**b**) 56 nm and (**c**) 106 nm with the respective d-spacing. The super-lattice given on (**d**) is clearly observed for the bigger nanoparticle, indicating a close behavior to their bulk counterpart. Vesta software was used to acquire the structural image [28].

Furthermore, a magnification reveals the fine crystalline feature of the structures and allows the measurement of the d-spacing, depicted on the inset of Figure 2b. For all samples, the diffraction plane related to the mean d-spacing corresponds to the (012) Miller index, along the *a*-axis. As given in Figure 3, there is an increase of the d${}_{hkl}$ values as the particle size expands from 26 nm to 58 nm. This evidence indicates that the nanostructures growth and the crystallization of LSMO occur along the *a*-axis. For the particular case of the 106 nm sample, the high resolution images give a clear visualization that the super-lattice is close to the observed for the bulk counterpart [27]. In addition, the obtained d-spacing for all samples is related to the La/Sr-La/Sr distances into the structures. It is worth to mention that the amorphous phases for the sample with 26 nm were confirmed from TEM analysis.

### 3.2. Magnetic Properties

**Figure 4:**
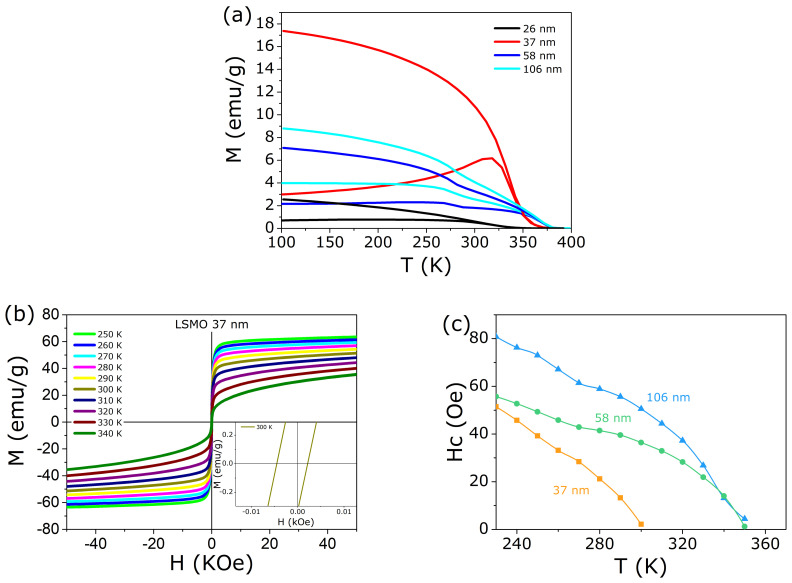
(**a**) ZFC and FC protocols for all samples prepared, with a clear difference for these two methods. It is a direct influence of the NP size on the magnetic properties of these compounds, even being of the same composition. (**b**) Magnetization as a function of magnetic field below the critical temperature, for several values of temperature. This result is representative for the other samples. Note the hysteresis (inset). (**c**) Coercive field as a function of temperature, for all samples prepared: from this result we determine the blocking temperature, as the one in which $H_{c}$ vanishes.

Field Cooled (FC) and Zero Field Cooled (ZFC) curves for the samples under consideration are shown in Figure 4a. Considering these samples are NPs and there is a remarkable difference for the ZFC and FC protocols, the corresponding blocking temperature must be determined. For this purpose, we measured the hysteresis curves for all samples below the critical temperature, as shown in Figure 4b. The critical temperature was obtained from the peak of the first derivative of the FC curve (not shown). From these isothermal measurements, we could obtain the coercive field as a function of temperature $H_{c}\left(T\right)$—see Figure 4c. The temperature in which the coercive field vanishes gives the blocking temperature [29,30].

**Figure 5:**
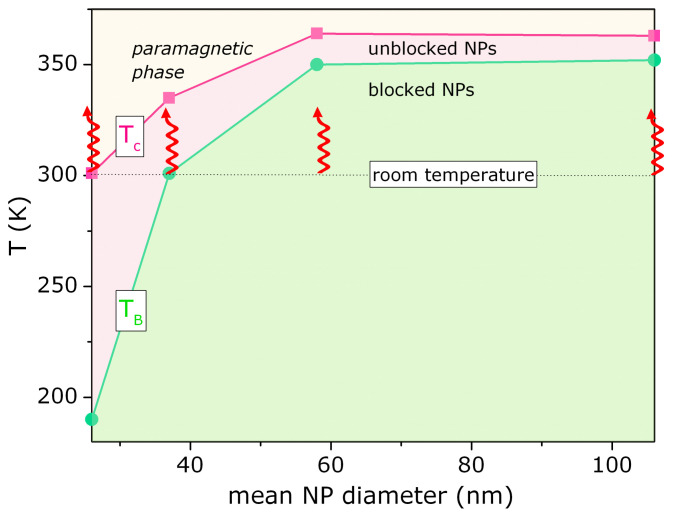
Phase diagram based on blocking and critical temperatures as a function of mean NP diameters for the samples prepared. Note the different regions in which the samples are heated up, upon hyperthermia experiment: region with magnetically blocked NPs (bigger NPs), paramagnetic NPs (smaller NPs) and magnetically unblocked NPs (intermediate size).

Figure 5 summarizes these experimental findings: the blocking and critical temperature as a function of the mean NP size, represented then as a phase diagram. Both characteristic temperatures increase monotonically, with well defined temperature intervals:

$T<T_{B}$ NP magnetic moments are blocked due to the anisotropic energy barrier;
$T_{c}>T>T_{B}$ NP magnetic moments are unblocked and free to rotate (*superparamagnetic* region);
$T>T_{c}$ There is no local magnetic ordering into the NP (*paramagnetic* region).

Note the room temperature is an important reference temperature, because the heating experiments start from this base point. Further discussion about this topic will be delivered further in this article.

### 3.3. Specific Absorption Rate—SAR

**Figure 6:**
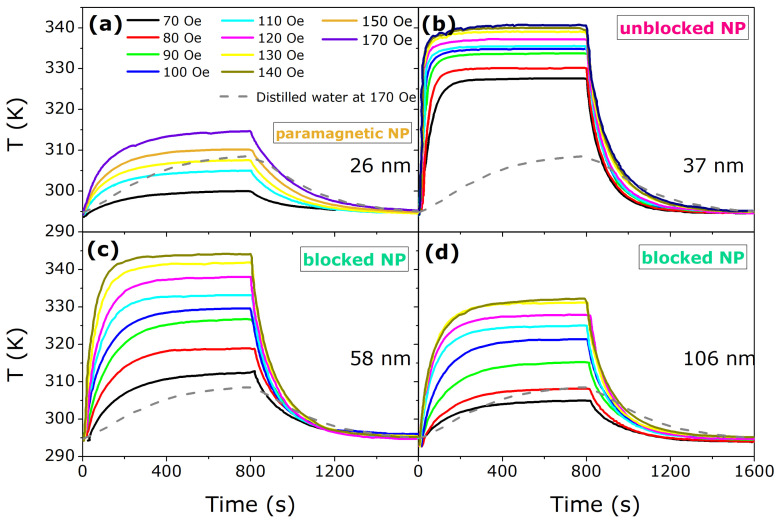
Heating-cooling curves for all samples prepared, for several values of AC-magnetic field. (**a**) presents the heating-cooling curves for sample 26 nm, (**b**) sample 37 nm, (**c**) 58 nm, and (**d**) 106 nm. The most efficient heating sample is the one with 37 nm of mean diameter size, because these are heated up, from room temperature, within magnetically unblocked region. See the phase diagram of Figure 5 and the text for further details.

Figure 6 illustrates the heating-cooling curves for the samples prepared, for several values of AC-magnetic field and the background tube with 100 mg of distilled water (dotted lines). These results have a direct connection with the phase diagram presented on Figure 5. Samples with bigger NPs size of 58 and 106 nm are heated up, from room temperature, within the magnetically blocked region (see the arrows in Figure 5). This region suffers from magnetic blockage due to the anisotropy. From that phase diagram, we also expect no heating efficiency for the sample with 26 nm, considering the heating curve runs within the paramagnetic region. For the sample with 37 nm, this is heated up within the magnetically unblocked region and therefore a maximum efficiency of heating is expected, as observed.

From these heating-cooling curves for all samples prepared, the Specific Absorption Rate-SAR could be obtained through the following relation:

(4) $$SAR=c\frac{m}{M}\frac{\Delta T}{\Delta t},$$

where *c* is the specific heat of the distilled water, *m* and *M* represent, respectively, the fluid and samples masses. In addition, ΔT/Δt is the initial slope of the heating curve [11], obtained from the heating-cooling curve, as detailed in the Section 2. In order to further explore this heating-cooling efficiency as a function of the NP size, we have looked upon the theoretical model for relaxation mechanisms of heating [31], where the dependence of the power dissipation with frequency can be described as follows:

(5) $$P=\pi \mu_{0}\chi_{0}H_{0}^{2}f\frac{2\pi f\tau }{1+{\left(2\pi f\tau \right)}^{2}}∼\frac{1}{2\pi \tau }\frac{{\left(\omega \tau \right)}^{2}}{1+{\left(\omega \tau \right)}^{2}}.$$

Above, *f* is the frequency of the radiation applied over the system, τ is the characteristic relaxation time of the magnetization, $\mu_{0}$ is the magnetic permeability of the vacuum, $\chi_{0}$ is the equilibrium magnetic susceptibility and $H_{0}$ the amplitude of the AC-magnetic field.

**Figure 7:**
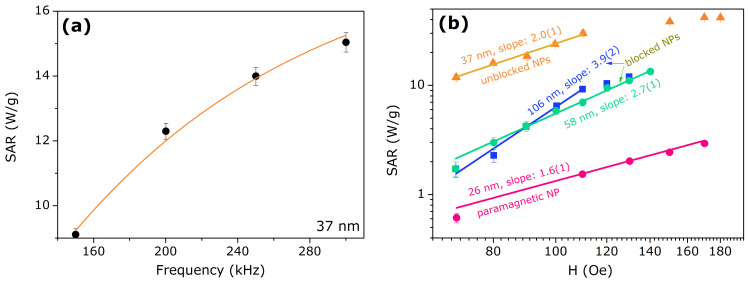
Specific Absorption Rate—SAR as a function of (**a**) frequency (sample with mean diameter size of 37 nm) and (**b**) magnetic field (all samples prepared). Solid lines represent the theoretical model (Equation (5)).

The frequency dependence for SAR in Figure 7a corresponds to the model of Equation (5), where the obtained fitting parameter was τ=0.9(1) μs, for the NP with 37 nm of mean diameter size. From this result and the model proposed by R. Rosensweig [31], considering this characteristic relaxation time τ, there are two concurrent heating mechanisms: Néel and Brown. This assumption is confirmed by the field dependence of the SAR measurement for this sample, presented in Figure 7b (log-log scale). From this figure, it is possible to obtain the corresponding slope, related to the exponent of the magnetic field (see Equation (5)). We found a quadratic slope of 2.0(1), as a confirmation for the Néel and Brown relaxations for this sample.

Smaller NPs, with mean diameter size of 26 nm, were heated up within the paramagnetic region and therefore the relaxation time is expected to be much faster than the reference case above described. Thus, for this limiting case, we expect τ≪1 μs and the ratio $\frac{{\left(\omega \tau \right)}^{2}}{1+{\left(\omega \tau \right)}^{2}}$ in Equation (5) tends to zero, minimizing the heat production. From the SAR measurement as a function of magnetic field, presented in Figure 7b, we found a slope of 1.6(1), representing a sub-quadratic exponent for the magnetic field.

Bigger NP of mean diameter size of 58 nm and 106 nm are heated up within the magnetically blocked region ($T<T_{B}$) and therefore we expect a slower relaxation time in comparison with the reference case (37 nm). Thus, for this limiting case, we expect τ≫1 μs and the ratio $\frac{{\left(\omega \tau \right)}^{2}}{1+{\left(\omega \tau \right)}^{2}}$ in Equation (5) tends to the unity. Consequently, for these larger NPs, the SAR values are smaller than the reference case due to the factor $\frac{1}{2\pi \tau }$. From Rosensweig’s model, this scale of relaxation time is dominated by Brown mechanism. However, another mechanism must also be taken into account: hysteresis, considering the heating process occurs for $T<T_{B}$ (see Figure 4b). This assumption is verified from the SAR measurement as a function of magnetic field, presented in Figure 7b, in which we found slopes of 2.7(1) and 3.9(2), for the samples of 58 nm and 106 nm, respectively. These values represent a super-quadratic exponent for the magnetic field (see Equation (5)), emphasizing these samples have an extra mechanism of heating (hysteresis), in addition to Brown relaxation.

Furthermore, to compare with the reported values in literature, Table 2 summarizes a few important parameters of coated and un-coated nanoparticles with potential for hyperthermia applications reported in the literature. As can be noted, Fe_3_O_4_ (magnetite) is among the systems with the greatest potential for several biomedical applications [32,33]. The type of coating is also important to improve the SAR values, where for the magnetite a 100× of enhancement is observed from the coating with silica to PEG for the [34]. Similarly, the Fe_2_O_3_ are also used for magnetic hyperthermia application due to its low toxicity, high saturation magnetization and tuned Curie temperature [34]. The presence of Mn on γ-Fe_2_O_3_ suppresses the transition to the antiferromagnetic α-Fe_2_O_3_, but there is a reduction on the magnetization. The antiferromagnetic suppression transition revealed to be efficient in the destruction of cells, with the disruption of the cytoskeleton [35]. Regarding nanostructures without the surface coating, the Gd_5_Si_4_ in its way has a $T_{C}$ near the therapeutic range that can be tuned so the heating would be self-regulated [1]. However, although Si-based materials are potentially biocompatible, the system cytotoxicity has not yet been evaluated [1,36]. As for the La_0.75_Sr_0.25_MnO_3_ nanoparticles, they present low toxicity, biocompatibility, and a high SAR value; however, they also tend to aggregate [11]. By increasing the particle size from 21 nm to 37 nm, which is a result of the present work, there is an increase in the SAR values from 23 to 30 W/g. Further increasing particle size, lead to a reduction in the SAR values, indicating a maximum due to the unblocked range of temperature. Thus, the heat transfer can be maximized due to the room temperature blocking temperature. In this regard, further experiments should be performed in LSMO nanoparticles aiming colloidal stabilization and functionalization aiming practical use of this system for magnetic thermal applications.

## 4. Conclusions

We have prepared nanoparticles (NPs) of lanthanum strontium manganites with the composition La_0.75_Sr_0.25_MnO_3_ by handling the annealing temperature of the samples. We obtained four samples, with mean NP diameter size ranging from 37 nm up to 106 nm—values obtained from Transmission Electronic Microscopy (TEM), with fine crystalline features.

Magnetization measurements reveal a remarkable ZFC-FC irreversibility; and an analysis of the coercive field as a function of temperature (below the critical temperature), for all samples produced, allows the determination of the blocking temperature. From these characteristic temperatures, we made a phase diagram for this system, as a function of mean NP diameter size. We observed that the smaller NP (26 nm) is heated up within the paramagnetic range of temperature ($T>T_{c}$), and for this reason, does not provide a substantial heating efficiency. The bigger NPs, on the other hand, are heated up, from room temperature, within the magnetically blocked range of temperature ($T<T_{B}$). For this reason, we observed a small heating efficiency—comparable to the case of small NPs. The main finding of this article is related to the heating process for NPs within the magnetically unblocked range of temperature ($T_{c}>T>T_{B}$), maximizing the efficiency of heat transfer. Further description of these arguments can be found in the main text of the article.

## Figures and Tables

**Table 1:** Crystallographic and morphological parameters for produced NPs, obtained from Rietveld refinement and TEM images. $T_{A}$ represents the annealing temperature; 〈D〉 and $D_{s}$ are, respectively, the mean NP diameter obtained from NP size distribution and Scherrer equation; *a*, *b* and *c* represent the lattice parameters, obtained from Rietveld refinements, while *V* is the unit cell volume.

Sample	$T_{A}$ (°C)	〈D〉 (nm)	$D_{s}$ (nm)	*a*, *b* (Å)	*c* (Å)	*V* (Å^3^)
1 *	600	26 (1)	24.0 (4)	5.4926 (1)	13.3597 (3)	349.04 (4)
2	800	37 (2)	32.7 (1)	5.4753 (2)	13.3441 (1)	348.97 (3)
3	900	58 (2)	30.3 (2)	5.45325 (4)	13.4611 (1)	346.67 (3)
4	1000	106 (4)	87.0 (1)	5.451 (1)	13.4598 (3)	346.51 (4)

* 88% amorphous.

**Table 2:** Summary of suitable parameters of nanoparticles from the literature with potential for hyperthermia applications: Diameter (*D*), type of coating, Curie temperature ($T_{C}$), blocking temperature ($T_{B}$), saturation magnetization ($M_{S}$) and specific absorption rate (SAR).

Compound	*D* (nm)	Coated	$T_{C}$ (K)	$T_{B}$ (K)	$M_{S}$ (emu/g)	SAR (W/g)	Reference
Fe_2_O_3_	19	Yes (PEG)	713	270	80	2452	[34]
Fe_3_O_4_	10	Yes (Silica)	713	200	3	20	[33,37]
γ-Fe_2_O_3_	5	Yes (Silica)	520	-	2.5	20	[38]
Gd_5_Si_4_	15	No	338	-	2	3.7	[1,36]
Cu_30_Ni_70_	30	No	315	-	1	12	[39]
${\mathrm{Mn}}_{1+x}{\mathrm{Fe}}_{2-2x}{\mathrm{Ti}}_{x}O_{4}$	20	No	318	250	8	2	[40,41,42]
La_0.75_Sr_0.25_MnO_3_	21	No	366	332	28	23	[11]
La_0.75_Sr_0.25_MnO_3_	26	No	300	190	4.8	5.6	This work
	37	No	330	303	46.1	30	This work
	58	No	370	350	25.9	6.9	This work
	106	No	370	350	35.9	9.3	This work

**Figure A1:**
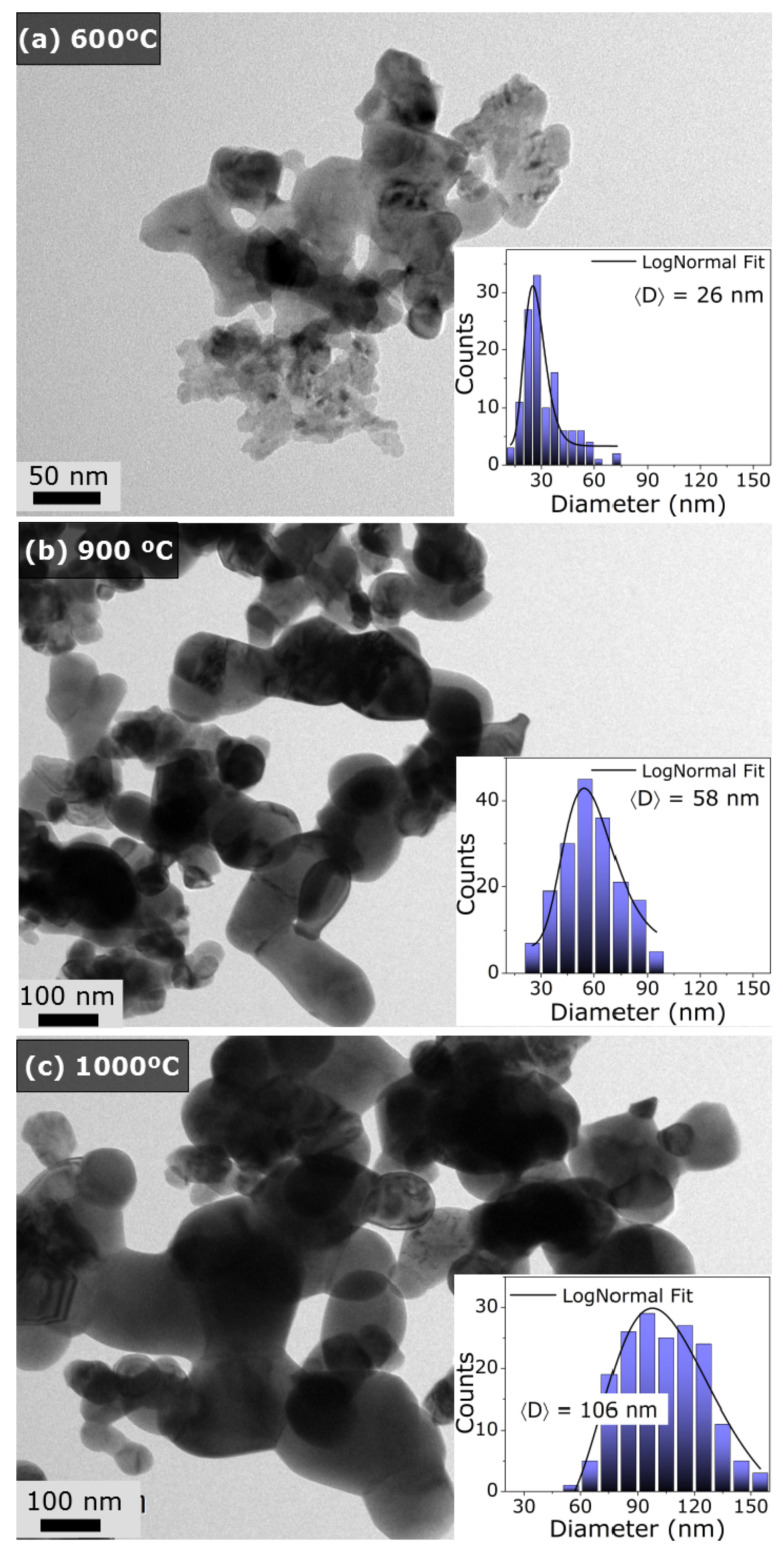
TEM images and particle size distribution for the samples prepared at (**a**) 600 °C (26 nm), (**b**) 900 °C (58 nm), and (**c**) 1000 °C (106 nm).

## Sample Availability

Samples of the La-Sr manganites of this work are available from the authors.

## Appendix A. Transmission Electronic Microscopy

Figure A1 presents complementary panels with Transmission Electronic Microscopy (TEM) for the samples annealed at 600 °C (26 nm), 900 °C (58 nm), and 1000 °C (106 nm). This figure also presents the NP counting and the corresponding LogNormal fitting. See the main text for further information.

## References

[1] Ahmad S., Akin Y., Shaheen S. (2005). Gd_5_(Si, Ge)_4_ and Gd_2_C compounds: Candidates for hyperthermia treatment of cancer. J. Appl. Phys..

[2] Astefanoaei I., Gimaev R., Zverev V., Stancu A. (2019). Modelling of working parameters of Gd and FeRh nanoparticles for magnetic hyperthermia. Mater. Res. Express.

[3] Deatsch A.E., Evans B.A. (2014). Heating efficiency in magnetic nanoparticle hyperthermia. J. Magn. Magn. Mater..

[4] Falk M., Issels R. (2001). Hyperthermia in oncology. Int. J. Hyperth..

[5] Haik Y., Chen C.J. (2011). Magnetic Particle Composition for Therapeutic Hyperthermia. U.S. Patent.

[6] Overgaard J. (1989). The current and potential role of hyperthermia in radiotherapy. Int. J. Radiat. Oncol. Biol. Phys..

[7] Herman T., Teicher B., Jochelson M., Clark J., Svensson G., Coleman C. (1988). Rationale for use of local hyperthermia with radiation therapy and selected anticancer drugs in locally advanced human malignancies. Int. J. Hyperth..

[8] Gilchrist R., Medal R., Shorey W.D., Hanselman R.C., Parrott J.C., Taylor C.B. (1957). Selective inductive heating of lymph nodes. Ann. Surg..

[9] Kumar C.S., Mohammad F. (2011). Magnetic nanomaterials for hyperthermia-based therapy and controlled drug delivery. Adv. Drug Deliv. Rev..

[10] Thiesen B., Jordan A. (2008). Clinical applications of magnetic nanoparticles for hyperthermia. Int. J. Hyperth..

[11] Pimentel B., Caraballo-Vivas R., Checca N., Zverev V., Salakhova R., Makarova L., Pyatakov A., Perov N., Tishin A., Shtil A. (2018). Threshold heating temperature for magnetic hyperthermia: Controlling the heat exchange with the blocking temperature of magnetic nanoparticles. J. Solid State Chem..

[12] Pękała M., Pękaa K., Drozd V., Fagnard J., Vanderbemden P. (2010). Magnetocaloric effect in La_0.75_Sr_0.25_MnO_3_ manganite. J. Magn. Magn. Mater..

[13] Manzoor S., Ahmed A., ur Rashid A., Ahmad S., Shaheen S. (2013). Study of magnetothermal properties of strontium doped lanthanum manganite nanoparticles for hyperthermia applications. IEEE Trans. Magn..

[14] Caraballo-Vivas R.J. (2017). Magnetism from intermetallics and perovskite oxides. arXiv.

[15] Shinde K., Pawar S., Pawar S. (2011). Influence of annealing temperature on morphological and magnetic properties of La_0.9_Sr_0.1_MnO_3_. Appl. Surf. Sci..

[16] Rodríguez-Carvajal J. (1993). Recent advances in magnetic structure determination by neutron powder diffraction. Phys. B Condens. Matter.

[17] Wildeboer R., Southern P., Pankhurst Q. (2014). On the reliable measurement of specific absorption rates and intrinsic loss parameters in magnetic hyperthermia materials. J. Phys. D Appl. Phys..

[18] Van Cuong P., Dho J., Park H.Y., Kim D.H. (2009). A sonochemical-assisted synthesis and annealing temperature effect of La_0.7_Sr_0.3_MnO_3_ nanoparticles. Appl. Phys. A.

[19] Murthy N., Minor H. (1990). General procedure for evaluating amorphous scattering and crystallinity from X-ray diffraction scans of semicrystalline polymers. Polymer.

[20] Andrade V.M., Vivas R.C., Pedro S.S., Tedesco J.C.G., Rossi A.L., Coelho A.A., Rocco D.L., Reis M.S. (2016). Magnetic and magnetocaloric properties of La_0.6_Ca_0.4_MnO_3_ tunable by particle size and dimensionality. Acta Mater..

[21] Qin H., Hu J., Chen J., Wang Y., Wang Z. (2002). Giant magnetoimpedance and colossal magnetoresistance in La_0.75_Sr_0.25_MnO_3_ at room temperature. J. Appl. Phys..

[22] Navin K., Kurchania R. (2018). The effect of particle size on structural, magnetic and transport properties of La_0.7_Sr_0.3_MnO_3_ nanoparticles. Ceram. Int..

[23] Hedayatnasab Z., Abnisa F., Daud W.M.A.W. (2017). Review on magnetic nanoparticles for magnetic nanofluid hyperthermia application. Mater. Des..

[24] Liu C., Zou B., Rondinone A.J., Zhang Z.J. (2001). Sol-gel synthesis of free-standing ferroelectric lead zirconate titanate nanoparticles. J. Am. Chem. Soc..

[25] Sato K., Li J.G., Kamiya H., Ishigaki T. (2008). Ultrasonic dispersion of TiO_2_ nanoparticles in aqueous suspension. J. Am. Ceram. Soc..

[26] Wolfram S., Gray T. (2021). Wolfram Alpha. www.wolframalpha.com/input/?i=log+normal+distribution.

[27] Thi N’Goc H.L., Mouafo L.D.N., Etrillard C., Torres-Pardo A., Dayen J.F., Rano S., Rousse G., Laberty-Robert C., Calbet J.G., Drillon M. (2017). Surface-Driven Magnetotransport in Perovskite Nanocrystals. Adv. Mater..

[28] Momma K., Izumi F. (2008). VESTA: A three-dimensional visualization system for electronic and structural analysis. J. Appl. Crystallogr..

[29] Guimarães A.P., Guimaraes A.P. (2009). Principles of Nanomagnetism.

[30] De Oliveira L., Pentón-Madrigal A., Guimarães A., Sinnecker J. (2016). Thermally activated processes and superparamagnetism in Bi_12_MnO_20_ nanoparticles: A comparative study. J. Magn. Magn. Mater..

[31] Rosensweig R.E. (2002). Heating magnetic fluid with alternating magnetic field. J. Magn. Magn. Mater..

[32] Shen L., Li B., Qiao Y. (2018). Fe_3_O_4_ Nanoparticles in Targeted Drug/Gene Delivery Systems. Materials.

[33] Le Renard P.E., Lortz R., Senatore C., Rapin J.P., Buchegger F., Petri-Fink A., Hofmann H., Doelker E., Jordan O. (2011). Magnetic and in vitro heating properties of implants formed in situ from injectable formulations and containing superparamagnetic iron oxide nanoparticles (SPIONs) embedded in silica microparticles for magnetically induced local hyperthermia. J. Magn. Magn. Mater..

[34] Guardia P., Di Corato R., Lartigue L., Wilhelm C., Espinosa A., Garcia-Hernandez M., Gazeau F., Manna L., Pellegrino T. (2012). Water-soluble iron oxide nanocubes with high values of specific absorption rate for cancer cell hyperthermia treatment. ACS Nano.

[35] Prasad N.K., Rathinasamy K., Panda D., Bahadur D. (2007). Mechanism of cell death induced by magnetic hyperthermia with nanoparticles of *γ*-Mn*_x_*Fe_2−*x*_O_3_ synthesized by a single step process. J. Mater. Chem.

[36] Nauman M., Alnasir M.H., Hamayun M.A., Wang Y., Shatruk M., Manzoor S. (2020). Size-dependent magnetic and magnetothermal properties of gadolinium silicide nanoparticles. RSC Adv..

[37] Elouafi A., Moubah R., Derkaoui S., Tizliouine A., Cherkaoui R., Shi S., Bendani A., Sajieddine M., Lassri H. (2019). Finite size effects on the magnetocaloric properties around blocking temperature in *γ*-Fe_2_O_3_ nanoparticles. Phys. A Stat. Mech. Its Appl..

[38] Owens F.J. (2009). Ferromagnetic resonance observation of a phase transition in magnetic field-aligned Fe_2_O_3_ nanoparticles. J. Magn. Magn. Mater..

[39] Kuznetsov A.A., Leontiev V.G., Brukvin V.A., Vorozhtsov G.N., Kogan B.Y., Shlyakhtin O.A., Yunin A.M., Tsybin O.I., Kuznetsov O.A. (2007). Local radio frequency-induced hyperthermia using CuNi nanoparticles with therapeutically suitable Curie temperature. J. Magn. Magn. Mater..

[40] Martirosyan K.S. (2012). Thermosensitive magnetic nanoparticles for self-controlled hyperthermia cancer treatment. J. Nanomed. Nanotechnol..

[41] Shimizu T., Asano H., Matsui M. (2007). Ferromagnetic exchange interaction and Curie temperature of Mg_1+*x*_Fe_2−2*x*_Ti*_x_*O_4_ (x = 0–0.5) system. J. Magn. Magn. Mater..

[42] Ferk G., Drofenik M., Lisjak D., Hamler A., Jagličić Z., Makovec D. (2014). Synthesis and characterization of Mg_1+*x*_Fe_2−2*x*_Ti*_x_*O_4_ nanoparticles with an adjustable Curie point. J. Magn. Magn. Mater..

